# Effectiveness of Telerehabilitation-Based Therapeutic Exercise on Functional Capacity in Chronic Stroke: Study Protocol for a Multicenter Randomized Controlled Trial

**DOI:** 10.3390/life15121905

**Published:** 2025-12-12

**Authors:** Yaiza Casas-Rodríguez, Carlos López-de-Celis, Gala Inglés-Martínez, Lidia González-Tova, María Benilde Martínez-González, Izaskun Barayazarra-López, Anna Escribà-Salvans

**Affiliations:** 1 Study Group on Pathology of the Locomotor System in Primary Care (GEPALAP), Jordi Gol i Gurina University Institute for Primary Health Care Research Foundation (IDIAPJGol), 08007 Barcelona, Spain; yaizacasas@gencat.cat; 2 Primary Health Care, Institut Català de la Salut, 08007 Barcelona, Spain; gingles.apms.ics@gencat.cat (G.I.-M.); lgonzalezt.apms.ics@gencat.cat (L.G.-T.); bmmartinez.apms.ics@gencat.cat (M.B.M.-G.); ibarayazarra.apms.ics@gentcat.cat (I.B.-L.); 3 Department of Physiotherapy, Faculty of Medicine and Health Sciences, Universitat Internacional de Catalunya Sant Cugat del Vallès, 08195 Barcelona, Spain; 4 ACTIUM Functional Anatomy Group, Faculty of Medicine and Health Sciences, Universitat Internacional de Catalunya, Sant Cugat del Vallès, 08195 Barcelona, Spain; 5 Research Group on Methodology, Methods, Models and Results of the Social and Health Sciences (M3O), Faculty of Health and Welfare Sciences, University of Vic-Central University of Catalonia (UVic-UCC), 08500 Barcelona, Spain; anna.escriba1@uvic.cat; 6 Center for Research in Health and Social Assistance (CESS), University of Vic-Central University of Catalonia (UVic-UCC), 08500 Barcelona, Spain; 7 Institute for Research and Innovation in Life and Health Sciences of Central Catalonia (IRIS-CC), University of Vic-Central University of Catalonia (UVic-UCC), 08500 Barcelona, Spain

**Keywords:** stroke, chronic disease, primary care, therapeutic exercises, telerehabilitation

## Abstract

**Background**: Stroke is the leading cause of physical disability in adults in Catalonia. Despite this, there is a lack of evidence of physiotherapy interventions on functional capacity during the chronic phase of the pathology. This multicenter clinical trial will be conducted with a sample size of 75 participants. **Objectives**: The objective of the study is to evaluate the effectiveness of a therapeutic exercise program in physiotherapy using telerehabilitation to optimize functional recovery and quality of life in people with chronic stroke, and to determine its impact on adherence to the exercise program. **Methods**: This is a multicenter randomized controlled trial. Three parallel groups will be compared, and two will undergo the same type of therapy. A control group (CG) will perform conventional intervention in primary care. There will be two experimental groups; (EG1) will perform document-guided therapeutic exercises at home and (EG2) will perform therapeutic exercises at home guided by a telerehabilitation program. The outcomes to be measured are degree of independence of a person in their activities of daily living, assessed by the Barthel Index, motor function, muscle tone of the affected limbs, muscle strength of the affected limbs, balance, gait efficiency, perception of musculoskeletal pain, perception of fatigue, risk of falls, perception of quality of life, and the perception of perceived subjective change after treatment. These outcomes will be evaluated at baseline (T0), at ten weeks (T1) (end of the intervention), and at 18 weeks (T2). The study duration per patient will be 18 weeks (a ten-week intervention, followed by an eight-week intervention follow-up). The analysis will be performed using a mixed linear model (ANOVA 3X3) and significance level *p* < 0.05.

## 1. Introduction

Stroke is the leading cause of physical disability in adults in Catalonia, Spain [1]. The incidence in Catalonia is 175 cases per 100,000 inhabitants, representing 11,500 new cases per year [2]. In Europe, a 34% increase in stroke cases is expected in the coming years [3]. In Catalonia, new cases represent an average cost to the health system of 315 million euros per year [4]. Furthermore, stroke accounts for EUR 1109 million per year in informal care costs [5]. The progress made in the treatment of the acute phase, through the Stroke Code, the management of patients in Stroke Units and reperfusion therapies in acute ischemic stroke, has improved the functional prognosis of surviving patients [6]. However, the available data on the physical and emotional health sequelae experienced by patients in the chronic phase [4] illustrate the significant impact on people’s health: 77% report poor perceived health; 55.7% have walking difficulties and 52.2% struggle with activities of daily living [7]; 71.6% experience constant pain [8]; and 40% present with limb spasticity [9].

At present, there is no evidence of the finiteness of the cerebral somatotopic organization [10]. On the other hand, the existing mechanisms for its reconfiguration and adaptation based on synaptic adaptation are described [11]. Studies have shown that, although the highest level of resilience is found in the subacute phase of the pathology, between 30 and 90 days post-stroke, the brain can also be reorganized through residual plasticity in the chronic phase even if the efficiency of neural network transfer is reduced [12,13,14]. Axonal sprouting and dendritic remodeling are key processes of neuronal resilience that continue to occur during rehabilitation, even beyond the first year after stroke [15]. Neurotrophins play a crucial role in neuroplasticity, neurogenesis, and neuroprotection of the Central Nervous System. The upregulation of neurotrophin expression contributes to the prevention and recovery of neurotrophin disorders. Therapeutic exercise is one of the tools physiotherapists use for enhancing the expression of neurotrophic factors in the motor regions of the brain during rehabilitation [15,16,17]. It is a comprehensive mechanism for addressing specific sequelae, the clinical situation, and therapeutic objectives, structuring the activity according to the optimal dosage of each patient [18].

The Rehabilitation Plan of Catalonia, Spain, an instrument that promotes a biopsychosocial approach to the person-centered care process [19,20], and indicates that the overall provision of rehabilitation care after having suffered a stroke must be continuous: community rehabilitation services must guarantee optimal rehabilitation, to achieve the best functional level and quality of life for the post-stroke patient. On the other hand, it establishes that outpatient and/or home-based rehabilitation for patients in the chronic post-stroke phase is indicated only in cases with a notable deterioration in functional status, or when a complication related to an underlying condition has occurred [19,20]. In practice, the system does not contemplate any intervention in the chronic post-stroke phase that guarantees continuity.

The study conducted by the Stroke Alliance for Europe reports that the rehabilitation received by stroke survivors in Europe is not intense enough, is too short, and often does not address the ongoing problems arising from the pathology [21]. In addition, it also states that long-term support is often non-existent [21]. The Spanish Society of Neurorehabilitation, in its proposal for a model for the care of acquired brain injury published in 2021, states that the duration of the necessary treatment should not be subject to time limitations but should be conditioned to the patient’s response to treatment and the possibilities of improvement of the patient after stroke [22].

To address these limitations, technology-based rehabilitation interventions have been developed in recent decades, showing promising results in improving functional mobility and independence in stroke patients, including telerehabilitation [23,24,25,26]. The use of technology promotes repetitive and task-specific training, active patient participation, integration of constructive and concurrent feedback, and accurate measurement of functional improvement [23]. The latest updates of the Clinical Practice Guidelines recommend the use of telerehabilitation as evidence of its benefit on quality of life [27].

The COVID-19 pandemic further revealed the advantages of telerehabilitation, such as greater accessibility, participation, co-responsibility and personalization of treatment [28,29]. However, the current model of stroke recovery in Catalonia is subject to time limitations that can lead to a worsening of the patient’s degree of disability and quality of life.

The objective is to evaluate the effectiveness of a therapeutic exercise program in physiotherapy using telerehabilitation to optimize functional recovery and quality of life in people with chronic stroke. In addition, the study aims to determine whether the telerehabilitation format improves adherence to the therapeutic exercise program compared with the paper-based delivery method.

## 2. Materials and Methods

### 2.1. Study Design and Setting

This study is an assessor-blinded, multicenter trial with a ten-week treatment period followed by a two-month follow-up. It follows the consensus-based core recommendations from the stroke recovery and rehabilitation expert group [18], thereby improving the study’s development and the SPIRIT statement [30]. This study has been approved by the Research Ethics Committee of the Institut d’Investigació en Atenció Primària Jordi Gol (IDIAP Jordi Gol) (num. 24/190-P). It has been registered at ClinicalTrials.gov (NCT06944197). Participants will be randomly allocated in a 1:1:1 ratio to the control group (CG) (*n* = 25) or the experimental groups (EG1) (*n* = 25); (EG2) (*n* = 25). Patients are being recruited by five primary care centers of the Southern Metropolitan Management of the Institut Català de la Salut (ICS) in Catalonia, Spain.

### 2.2. Recruitment and Blinding

Participants will be randomly assigned to one of the three intervention groups using a computer-generated sequence through Random.org. To ensure allocation concealment, only the principal investigator (PI) will have access to the randomization list. This allocation will take place after obtaining informed consent, but before baseline assessments (T0), and the allocation will not be disclosed to the physiotherapists or outcome assessors until the participant has been formally enrolled in the study.

Although no stratification or block randomization procedures were applied, rigorous measures have been implemented to minimize potential bias and ensure the internal validity of the study. Outcome assessments will be performed by independent, previously trained physiotherapists acting as blinded assessors. In addition, statistical analyses will be conducted by researchers blinded to group allocation, thereby strengthening the objectivity of the results. All data will be recorded in a secure digital system, password-protected and accessible only to authorized members of the research team.

Prior to the randomization process, participant recruitment will follow a series of steps designed to ensure ethical transparency and informed understanding. First, the principal investigator, together with the physiotherapist responsible for each assigned center, will provide detailed information to collaborating healthcare professionals regarding the inclusion and exclusion criteria, as well as the general characteristics of the study. Second, physiotherapists from the research team will contact potential participants by phone through their primary care center to explain the purpose, design, duration, and potential risks and benefits of the study. If a patient expresses interest, a face-to-face appointment will be scheduled to provide more detailed information and resolve any questions. Informed consent will be obtained during this appointment, prior to accessing the participant’s medical history, in compliance with ethical standards and data privacy regulations.

**Figure 1:**
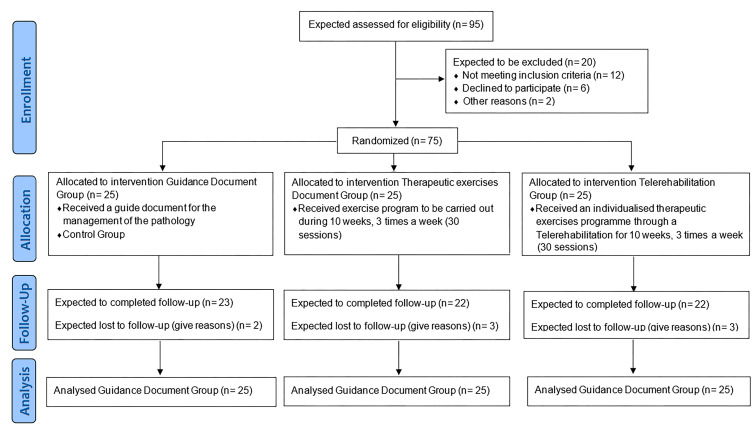
Planned Consort flow diagram: This diagram represents the planned participant flow; all “planned *n*” values will be replaced with actual numbers upon trial completion.

After group allocation and before starting treatment (T0), pre-intervention tests will be conducted to assess the baseline values of the primary and secondary outcome measures. A maximum of one week after completion of the intervention (T1), data will be collected for all efficacy outcomes (see Section 2.5). The same data will be collected two months after the end of the intervention (T2) (Figure 1). During the ten weeks of intervention, data will be collected in each session regarding adherence to the intervention (number of sessions and duration), intensity, exercises performed by each patient, and any incidents. All visits and efficacy evaluations will be conducted at the patient’s reference primary care center with the corresponding evaluating physiotherapist.

The study uses a single-blind design due to the nature of the interventions; therapists and participants cannot be blinded to treatment allocation. To avoid detection bias, efficacy outcomes will be assessed by an independent, blinded evaluator. Each center has a trained physiotherapist assessor, supervised by the principal investigator. The training information is available online and in paper format. Additionally, all statistical analyses will be conducted by researchers blinded to group allocation.

All data will be recorded in an online document shared by all the evaluators of the research team. A form created in a spreadsheet will be used. All researchers are trained in the use of the scales and the document, and they have a help guide, as well as a consultation service directly with the principal investigator of the study who is responsible for the logistics of the study. Access to the study registration document is restricted to authorized study personnel via password.

Furthermore, all data management procedures comply with the General Data Protection Regulation (GDPR) and the information security policies of the Institut Català de la Salut (ICS). Study data will be stored on ICS institutional servers, which implement standardized security and access-control measures. Access to the study data will be restricted to authorized research personnel using institutional credentials. Personal identifying information is not included in the study’s analytical database; instead, it is kept separately within ICS systems, and each participant is assigned a unique study identification code to ensure pseudonymisation. Remote and digital data collection is carried out using secure institutional platforms that guarantee data confidentiality and integrity throughout the study.

### 2.3. Sample Size

The sample size was calculated using an a priori power analysis conducted with G*Power software (version 3.1.9.7). The calculaion was based on a mixed-design repeated-measures ANOVA with one between-subjects factor (three groups) and one within-subjects factor (three time points). A conservative medium effect size (f = 0.32) was selected for the calculation, which represents a smaller effect than that reported by Aphiphaksakul et al. [31], who reported functional improvements following a mobili-application-based training protocol in stroke patients. This effect size corresponds to an expected between-group difference of approximately 8–10 points on the Barthel Index, substantially exceeding the established MCID of 1.85 [32,33], thereby ensuring clinically meaningful differences. The analysis employed a significance level of α = 0.05 and a statistical power of 0.80 (1 − β = 0.80), and 10% dropout rate. Based on these parameters, the required sample size was determined to be 75 participants, with 25 participants allocated to each group.

### 2.4. Selection Criteria

Patients will have to meet the following eligibility criteria to be included in the study. Inclusion criteria include being 18 years of age or older, having a medical diagnosis of stroke, and having had the condition for more than 6 months. Participants must have physical sequelae secondary to stroke, with a minimum score of 1 on the Medical Research Council scale for overall muscle strength of affected limbs, a maximum score of 3 on the Modified Ashworth scale for overall muscle tone of affected limbs, and a maximum score of 4 on the Modified Rankin scale for disability. Additionally, participants should have access to computer equipment, such as a tablet, desktop or laptop computer, or smartphone.

Exclusion criteria include undergoing other non-pharmacological physical treatments for the improvement and/or maintenance of stroke sequelae, previous decreased motor functional capacity with a Barthel scale score of less than 60 points, and cognitive and/or language barriers that hinder the correct understanding of the informed consent and intervention. Additionally, participation in another clinical trial during this study period that may impact the results of the variables to be evaluated is also an exclusion criterion.

During the study, participants may continue their usual daily physical activity (e.g., walking or household activities), but they may not engage in any additional structured physiotherapy, private rehabilitation, or therapeutic group sessions, as these could influence the study outcomes. To monitor possible concomitant therapies, participants will complete a weekly activity log, and the research physiotherapists will verify this information during each follow-up contact.

### 2.5. Outcome Measure

Descriptive data of the sample will be recorded, as well as anthropometric data and various assessment measures for the following evaluation variables.

The primary outcome is the degree of dependence or functional capacity for activities of daily living, which is assessed using the Barthel Index [32], and it is the validated scale used for the sample calculation [31].

The secondary outcomes have been classified into two categories: (1) Key Secondary Outcomes, which include motor function, muscle tone, muscle strength, balance, and quality of life, and (2) Additional Secondary Outcomes, which include gait performance (gait pattern and gait speed), perceived pain, fatigue, fall risk, subjectively perceived change after treatment, and adherence to the exercise program.

The secondary variables and their respective evaluation tools are as follows: motor function will be assessed using the Modified Motor Assessment Scale; muscle tone of the affected limbs will be measured with the Modified Ashworth Scale; muscle strength of the affected limbs will be evaluated using the Medical Research Council scale; balance will be assessed with the Postural Assessment Scale for Stroke Patients; walking efficiency will be measured using the Wisconsin Gait Scale and the 4 Meter Gait Speed Test; perception of osteomuscular pain will be evaluated through the Brief Pain Inventory; perception of fatigue will be measured using the Fatigue Severity Scale; fall risk will be assessed with the Time Up and Go Test; quality of life perception will be evaluated using the Newcastle Stroke-Specific Quality of Life Measure (Spanish version); and perception of subjective change post-treatment will be measured with the Global Rating of Change Scale (Table 1).

Adherence, included as an Additional Secondary Outcome, will be quantified as the proportion of prescribed sessions completed, the weekly frequency achieved, and the duration of each session, allowing further analysis of its potential influence on the functional outcomes observed across the intervention groups.

Although this broad set of outcomes aims to provide a comprehensive clinical profile of participants, the assessment burden is minimized through efficient scheduling of evaluation visits, grouping compatible measures within the same session, and using validated instruments with short administration times.

Given the number of secondary outcomes, results will be interpreted cautiously, and multiplicity will be addressed using appropriate statistical adjustments, as detailed in Section 2.8.

#### Assessments

The Barthel Index is a well-established and widely used tool for assessing physical disability, particularly in stroke patients. It consists of 10 items that evaluate personal care and mobility, yielding a total score ranging from 0 (totally dependent) to 100 (independent) [33].

The Modified Motor Assessment Scale (MMAS) is a valid and reliable instrument used to assess motor function in stroke patients, focusing on limb and trunk mobility. Scoring ranges from 0 to 5 for each item, with higher scores indicating better function. This tool demonstrates high interobserver and test–retest reliability, along with strong construct and concurrent validity [34,35].

The Modified Ashworth Scale (MAS) is widely used for assessing muscle tone and spasticity in stroke patients. It scores spasticity from 0 (no spasticity) to 4 (severe spasticity). Its construct and concurrent validity are strong, confirming its utility in evaluating muscle tone and spasticity [36,37].

The Medical Research Council (MRC) Scale is a well-regarded tool for assessing muscle strength in stroke patients, scoring from 0 (no muscle contraction) to 5 (normal strength against gravity). The scale demonstrates good interobserver and test–retest reliability, along with solid construct and concurrent validity [38,39,40].

The Postural Assessment Scale for Stroke Patients (PASS) evaluates postural control and balance, focusing on a patient’s ability to maintain specific postures. It consists of 12 items, each scored from 0 to 3, where higher scores indicate better postural control. The PASS exhibits high reliability and predictive validity [41].

The Wisconsin Gait Scale (WGS) is a comprehensive tool for assessing gait, including postural control, stride length, and gait symmetry. It is scored from 0 to 3, with higher scores reflecting better gait function. The WGS has high interobserver and test–retest reliability, along with strong concurrent and predictive validity [31].

The 4-Meter Gait Speed Test assesses walking speed, a crucial measure of mobility, independence, and fall risk. The test is highly reliable for evaluating functional prognosis and rehabilitation progress [42].

The Brief Pain Inventory (BPI) measures pain intensity and its impact on quality of life, using a scale from 0 (no pain) to 10 (worst pain imaginable). The BPI is widely used due to its strong reliability and validity [43].

The Fatigue Severity Scale (FSS) evaluates the severity of fatigue and its impact on daily activities, with scores ranging from 1 (strongly disagree) to 7 (strongly agree). The FSS is highly reliable for monitoring fatigue in stroke rehabilitation [44].

The Timed Up and Go (TUG) Test assesses fall risk by timing how long it takes for a patient to stand up from a chair, walk 3 m, turn, return, and sit down. The TUG test is a reliable and valid measure for fall risk [45].

The Newcastle Stroke-Specific Quality of Life Measure (NEWSQOL) evaluates stroke-specific quality of life, encompassing physical, emotional, social, and cognitive domains. It has demonstrated high internal reliability and strong construct, concurrent, and predictive validity [46].

The Global Rating of Change Scale (GROC) measures the patient’s perception of changes in their health or functioning after an intervention. It demonstrates strong test–retest reliability and robust concurrent and construct validity [47].

Information regarding stroke diagnosis, medical history, and stroke onset will be collected from patient records in the ECAP programs (ICS). Additionally, data on participant characteristics, including sex, age, family support, primary caregiver involvement, medication use, comorbidities, dysphagia, lesion location and side, time post-stroke, and stroke severity (assessed by the NIHSS and modified Rankin Scale), will be gathered for comprehensive analysis.

### 2.6. Interventions

#### Intervention Description

The sample will be randomized systematically and pseudonymized. The 75 subjects will be divided into three intervention groups, and a different intervention will be carried out in each of them:

- Guidance Document Group (*n* = 25): The primary and community care physiotherapist will carry out an individual face-to-face consultation at the Primary Care Center with the person participating in the study, where they will deliver a guide document for the management of the pathology, as well as guidelines for the promotion and prevention of important health issues after a stroke. The document is the “Guide aimed at people affected by cerebral vascular disease and their families and caregivers” created by the Department of Health of the Generalitat de Catalunya, together with the Stroke Foundation in 2022. This group is the control, since the intervention is the one that most resembles the intervention that is currently being carried out in Catalonia from the Primary Care Centers for Stroke in the chronic phase.

No additional therapeutic contacts are scheduled after this initial session, and no monitoring is performed, as this reflects current standard practice in Catalonia.

No adherence tracking applies in this group, as no structured exercise program is prescribed.

- Therapeutic Exercises Document Group (*n* = 25): The physiotherapist will carry out an individual face-to-face consultation at the Primary Care Center with the person participating in the study. The document “Home-Based Therapeutic Exercise Program” will be explained and delivered, and contains an exercise program to be carried out during the next 10 weeks, with a frequency of 3 times a week. The explanatory document consists of 3 exercise tables for each week of intervention (30 sessions) [48,49,50,51,52]. In addition to the corresponding description of each exercise for the correct performance, it will also specify the dosage, both in repetitions, as well as in series and load to be performed. It will also contain a checklist template for tracking completed activities. Strength exercises will be performed for the different muscle groups of the lower extremity and upper extremity, and trunk. Also, stretching exercises for the most relevant flexor muscles of the lower extremity and upper extremity and trunk will be undertaken. In addition, stretching exercises will be included for the most relevant flexor muscles of these regions. In addition, exercises wil be added for the efficiency of standing walking, such as the previous step. And, finally, mobility exercises and global balance will be included.

Although the exercise dose is identical to that of the telerehabilitation group (30 sessions over 10 weeks), participants in this group will also receive structured follow-up contacts. A brief telephone call (approximately 10–15 min) will be scheduled every two weeks (five contacts in total) to review adherence, address questions regarding the exercises and, when necessary, adjust the dosage or difficulty according to predefined criteria. Any additional clarification requested by participants will follow the usual communication channels of the primary care center, but no structured monitoring is performed.

Participants in this group will self-record adherence using the checklist provided within the exercise program.

- Telerehabilitation Group (*n* = 25): The physiotherapist will carry out an individual face-to-face consultation at the Primary Care Center with the person participating in the study, where the intervention of an individualized program of therapeutic exercises will be explained through a Telerehabilitation platform (Physitrack) for the following 10 weeks, with a frequency of 3 times a week (30 sessions). In addition, participants will be given an explanatory document called “Guide for the use of the Physitrack Telerehabilitation Platform” that contains the necessary information for the correct access and use of Physitrack. Likewise, the consultation will include a brief overview of the platform’s operation and a practical demonstration. The therapeutic exercise program will follow the same standardized structure and dosage as that of the Therapeutic Exercises Document Intervention Group (same exercices, number of sessions, sets, repetitions, intensity, and progression criteria). Participants in this group will receive structured follow-up every two weeks (five contacts in total), delivered through the telerehabilitation platform. These remote contacts (approximately 10–15 min each) will allow the physiotherapist to review adherence, address questions, and adjust the exercise program according to each patient’s progression. Participants will also be able to communicate directly with the physiotherapist through the platform.

In both the Therapeutic Exercises Document Group and the Telerehabilitation Group, the initial therapeutic exercise program is standardized. Each session includes approximately 8–10 exercises targeting the lower limbs, upper limbs, and trunk, combining strengthening, stretching, mobility, and balance tasks. Unless otherwise indicated, exercises are prescribed in 2–3 sets of 10–15 repetitions, at a moderate intensity guided by perceived exertion (11–13 on the Borg scale), with 1–2 min of rest between sets. Progression is applied every two weeks according to predefined criteria (e.g., increasing repetitions, sets, or load, or introducing a more challenging variation) when the patient is able to complete the exercises with good technique and without excessive fatigue or pain. A detailed description of the exercises and progression criteria is provided in Supplementary Table S1.

To ensure consistency across intervention groups, participants will be instructed to maintain only their usual daily physical activity during the study period. No additional structured physiotherapy, private rehabilitation, or external therapeutic exercise programs will be permitted, as these could influence the outcomes of the assigned intervention. Compliance with these instructions will be monitored through a weekly activity log completed by participants and reviewed by the physiotherapists, allowing the identification of any concomitant activities.

In addition, with the aim of analyzing the actual dose of treatment received, adherence will be assessed in all intervention groups based on the proportion of prescribed sessions completed, the weekly frequency achieved, and the duration of each session. These parameters will be systematically recorded to determine the effective exercise dose performed by each participant and to explore its potential influence on functional outcomes.

Finally, once the intervention period and the two-month follow-up evaluation (T2) have been completed, if the Telerehabilitation Group demonstrates superior results compared with the other two groups, participants from those groups will be offered the opportunity to receive an individualized therapeutic exercise program through the Physitrack platform. This measure ensures that all participants may benefit from the most effective therapeutic option, thereby upholding the ethical principles of equity and beneficence in research.

### 2.7. Safety Monitoring and Adverse Events

To ensure participant safety throughout the intervention period, all adverse events (AEs) occurring during the study will be systematically monitored, documented, and reported in accordance with institutional guidelines and research ethics standards.

Expected adverse events in the context of a therapeutic exercise program for individuals with chronic stroke may include mild musculoskeletal pain, transient fatigue exacerbation, dizziness, or accidental falls. Although these events are considered low risk, any occurrence will be recorded in detail and reviewed by the study physiotherapists.

An adverse event will be documented when a participant reports new symptoms, an exacerbation of pre-existing symptoms, or any physical incident occurring during or after an exercise session. The participant’s designated physiotherapist will record the event in the document specifically created for this purpose within the study’s secure system, including the date, description, severity, actions taken, and whether the participant required additional medical evaluation. Serious adverse events (SAEs), such as injuries requiring medical attention or hospital admission or any incident that compromises participant safety, will be reported immediately to the principal investigator.

All SAEs, as well as any adverse events judged to be related to the intervention, will be reported to the Research Ethics Committee (CEI) in accordance with institutional and regulatory requirements. The CEI will be informed within the timelines established by the institution’s protocols. Participants experiencing an adverse event will be assessed and, if necessary, may be temporarily or permanently withdrawn from the intervention to ensure their safety.

Regular safety monitoring will be carried out throughout the study by the principal investigator, who will oversee adherence to reporting procedures and ensure that all safety-related information is appropriately reviewed and managed.

### 2.8. Statistical Analysis

The statistical analysis will be performed with the IBM SPSS v.26.0 software. Descriptive statistics will be calculated for all variables. To check the normal distribution of the variables, the Kolmogorov–Smirnov test with the Lilliefors correction will be used. For the comparison of the groups, a mixed 3 × 3 ANOVA will be carried out with interaction time (three measurement time points) and group (three intervention groups). The assumption of sphericity will be checked by means of the Mauchly test; if it is not met, the Greenhouse–Geisser correction will be used for interpretation.

The primary outcome and the Key Secondary Outcomes (motor function, muscle tone, muscle strength, balance, and quality of life) will be analyzed following this main model, given their clinical relevance. Additional Secondary Outcomes (gait performance, perceived pain, fatigue, fall risk, and subjective perceived change) will be analyzed using the same statistical approach but interpreted with greater caution due to their supplementary nature.

When a statistically significant effect is observed, the post hoc test will be performed using the Bonferroni correction to adjust for multiple comparisons. This correction will be applied systematically to all post hoc analyses to address the multiplicity of secondary outcomes.

For qualitative variables, the Chi-square test or Fisher’s Exact statistic will be used. The effect size will be calculated using Cohen’s d. Cohen’s coefficients [53] are interpreted as follows: large effect sizes, d > 0.8; moderate effect sizes, d = 0.5–0.79; and small, d = 0.2–0.49. The significance level was established at *p* < 0.05 with a 95% confidence interval. Missing data will be handled using Little’s MCAR test and multiple imputation (MICE) performed separately for each intervention group using the SPSS multiple imputation module. The analysis will be undertaken on an “intent-to-treat” basis.

Adherence outcomes (percentage of sessions completed, weekly frequency, and session duration) will be analyzed descriptively and compared between groups. If appropriate, exploratory analyses (e.g., correlation or regression models) will be conducted to examine the potential mediating or moderating role of adherence in relation to functional outcomes.

## 3. Discussion

Currently, a 34% increase in the absolute number of strokes is expected by 2035 in Europe, as well as a 25% increase in the number of survivors living with the long-term effects of stroke. Therefore, it is not only necessary to take more efficient measures to prevent stroke, but it is also essential to ensure timely and appropriate treatment and rehabilitation to those who require it, with the aim of improving the lives of stroke survivors and their families by providing the appropriate level of support in their post-stroke life.

However, the current rehabilitation model for physiotherapeutic treatment in the chronic phase of stroke is subject to a temporal limitation without follow-up. This situation leads to the inability to achieve the capacity goals set between the physiotherapist and the patient at the beginning of treatment before it is completed. This has clinical implications for the functional status of the patients. Furthermore, there is no possibility of continuity without deterioration in the functional capacity over the long-term course of the condition in the patient.

This study will help to understand the effects of a 10-week therapeutic physiotherapy exercise program using telerehabilitation on the degree of dependence or functional capacity (Barthel Index) as the primary outcome. It is important to consider the evaluation of the degree of dependence and the functional outcomes of activities of daily living to determine whether the intervention is effective, which is why the Barthel Index was chosen. The minimally clinically important difference in the Barthel Index is set at approximately 1.85 points [32]. Furthermore, it will also be important to assess other measures, such as motor function of the limbs through the Modified Motor Assessment Scale (MMAS), to evaluate the progression or regression of motor skills over time and to guide treatment planning.

An abnormal increase in muscle tone is also an important condition to assess, using the Modified Ashworth Scale (MAS), as patients may experience spasticity in the muscles affected by brain injury, which can complicate the recovery of mobility and motor function. Additionally, the muscle strength of the four limbs will be measured using the Medical Research Council scale. The evaluation will have clinical relevance if the results show score changes of 2 points for the MMAS [54], and between 0.45 and 0.73 points in the lower extremities and between 0.48 and 0.76 points in the upper extremities for the MAS [55].

Gait difficulties are among the most common physical impairments after a stroke. The gait pattern is characterized by being asymmetric, with reduced speed and shortened stride length, which generally requires high energy expenditure. Improvement of the parameters will be considered when there is an increase of 2.5 points on the WGS scale [56], and approximately 0.09 m/s increase in baseline speed on the 4MWT [42]. Balance training, both dynamic and static, becomes essential to improving core functionality, as well as performing therapeutic exercises that consider the biomechanics of the trunk and lower limbs for potential improvement in gait quality. For its evaluation, the Postural Assessment Scale for Stroke Patients (PASS) and the Timed Up and Go (TUG) test have been chosen to assess fall risk. A positive evaluation at the end of the study will be considered if there is an improvement of 3 points on the PASS [57] scale and a change from 1.0 to 1.5 s in the TUG [58].

Assessing parameters that affect the daily lives of post-stroke patients is also highly relevant for evaluating the effectiveness of the study, such as measuring the perception of musculoskeletal pain using the Brief Pain Questionnaire and the perception of fatigue using the Fatigue Severity Scale.

It is also necessary to evaluate participation outcomes and quality of life [59]. Therefore, the scales used to measure the perception of quality of life will include NewsQol, and the Global Rating of Change Scale will be used to measure the perceived subjective change post-intervention.

The scales selected to evaluate the variables in this study have demonstrated good reliability and validity in stroke populations [60].

### Limitations

As with many physiotherapy intervention studies, therapist blinding was not feasible due to the nature of the treatment. Although this represents a methodological limitation, efforts were made to minimize bias through strict adherence to the predefined intervention protocol. Acknowledging this constraint highlights the importance of transparency and rigor in the design and interpretation of clinical trials in physiotherapy.

Another limitation of the present study is the unequal level of therapeutic support across the intervention groups. Although the two experimental groups receive the same exercise program and equivalent structured fortnightly follow-up, the control group receives only a single initial session without additional monitoring, as this reflects the current standard of care in Catalonia. This lower level of therapeutic engagement may influence motivation, adherence, and perceived support, potentially contributing to differences in outcomes beyond the specific intervention format. This factor should be considered when interpreting the results, and future studies may explore strategies to harmonize the amount of therapeutic contact across all study arms.

Moreover, this limitation underscores the need to complement quantitative findings with objective outcome measures and to consider triangulation strategies—such as using blinded assessors and standardized assessment tools—to enhance the credibility of the results. Future studies should continue to address this challenge by incorporating robust methodological safeguards that strengthen internal validity despite the impossibility of blinding therapists.

## 4. Conclusions

Chronic stroke patients with a stable clinical condition but residual functional disability require a continuous, individualized rehabilitation approach adapted to the different stages of their lives. Rehabilitation care should not only focus on initial recovery but also on preventing progressive functional decline and secondary conditions associated with physical inactivity and disability. In this context, therapeutic exercise is established as a fundamental tool for maintaining long-term functional autonomy and quality of life.

The results of this study may provide relevant evidence on the effectiveness of telerehabilitation as a complementary or alternative therapeutic modality to traditional in-person care. If proven effective in improving key functional variables—such as the ability to perform activities of daily living, muscle strength, balance, and perceived quality of life—this strategy could be integrated into Primary Care services. This would allow for expanded access to rehabilitation programs, particularly in rural or resource-limited areas, thereby promoting equitable, sustainable, and patient-centered care.

However, potential barriers to implementation in Primary Care must be acknowledged. These include the need for adequate technological infrastructure, sufficient digital literacy and training for both clinicians and patients, and the variability in resource availability across regions, particularly in rural settings. Additionally, reimbursement policies and organizational models may need adaptation to incorporate remote rehabilitation within existing service frameworks. Despite these challenges, the findings of this study may help guide future implementation strategies by identifying the conditions under which telerehabilitation can be safely, feasibly, and effectively deployed in real-world Primary Care environments.

## Figures and Tables

**Table 1:** Outcome measures and assessment tools.

Assessment Tools	Variables
Primary outcome	
Barthel Index	Functional capacity
Key Secondary Outcome	
Modified Motor Assessment Scale	Motor function
Modified Ashworth Scale	Muscle tone
Medical Research Council Scale	Muscle strength
Postural Assessment Scale for Stroke Patients	Balance
NewsQol	Perceived quality of life
Additional Secondary Outcome	
Wisconsin Gait Scale	Gait
4-Metre Gait Speed Test	Gait speed
Brief Pain Inventory	Perceived pain
Fatigue Severity Scale	Fatigue
Time Up and Go Test	Fall risk
Global Rating of Change Scale	Subjective perceived change
Adherence metrics	Exercise program adherence

## Data Availability

Not applicable.

## Supplementary Materials

The following supporting information can be downloaded at: https://www.mdpi.com/article/10.3390/life15121905/s1, Table S1: Representative Examples of the Multicomponent Therapeutic Exercise Program and Progression Criteria.

## Abbreviations

The following abbreviations are used in this manuscript:

CG	Conventional Group
EG	Experimental Group
BI	Barthel Index
MMAS	Modified Motor Assessment Scale
MAS	Modified Ashworth Scale
MRC	Medical Research Council
PASS	Postural Assessment Scale for Stroke Patients
WGS	Wisconsin Gait Scale
4MWT	4 Meter Gait Speed Test
BPI	Brief Pain Inventory
FSS	Fatigue Severity Scale
TUG	Time Up and Go Test
NEWSQOL	Newcastle Stroke-Specific Quality of Life Measure
GROC	Global Rating of Change Scale
SERNS	Spanish Society of Neurorehabilitation
RCT	Randomized Control Trial
ICS	Institut Català de la Salut
AE	Adverse events
