# Supplementary material for: Effectiveness of Telerehabilitation-Based Therapeutic Exercise on Functional Capacity in Chronic Stroke: Study Protocol for a Multicenter Randomized Controlled Trial
Source: Life (Basel). 2025 Dec 12;15(12):1905. doi: 10.3390/life15121905 (PMC12735140; doi:10.3390/life15121905)
Supplement: Supplementary file 1 [file life-15-01905-s001.zip › life-4034764-supplementary.pdf]

Supplementary Table S1. Representative Examples of the Multicomponent Therapeutic Exercise Program and Progression Criteria

Exercise Category	Example Exercises	Description (Summary)	Baseline Dosage	Progression Criteria	Progression Options
A. Strengthening Exercises – Lower Limb	Step-up and over; Sit-to-stand variations; Forward lunges	Strengthening of hip, knee, and ankle musculature; functional sit-to-stand patterns; unilateral loading on affected side	2–3 sets × 8–12 reps	Performs repetitions with good alignment and control; no loss of balance or compensations	Increase step height; add weights; increase repetitions or sets
B. Strengthening Exercises – Upper Limb	Weighted forward raise; Shoulder abduction with band; Wall push-ups	Upper limb strengthening targeting deltoid, scapular stabilizers, biceps/triceps; unilateral or bilateral tasks	2–3 sets × 10–15 reps	Full range of motion without pain; symmetrical effort; adequate scapular control	Increase resistance band level; add weights; increase repetitions
C. Core Stability	Bridge variations; Bird-dog; Side bridge; Seated trunk rotations	Activation and strengthening of abdominal and lumbar stabilizers; control of pelvis and trunk in static/dynamic tasks	2–3 sets × 8–12 reps or 10–15 sec holds	Maintains neutral spine; completes task without fatigue or compensatory patterns	Increase hold time; increase lever arm; add unstable surfaces
D. Balance Training	Tandem stance; Single-leg stance (supported); Weight-shifting; Step-ups	Static and dynamic balance to improve postural control, ankle/hip strategies, and anticipatory reactions	2 sets × 30–45 sec (static) or 10–12 reps (dynamic)	Able to maintain posture without sway or assistance	Reduce hand support; increase time; add perturbations; increase surface instability
E. Mobility / Gait Training	Marching in place; 4-meter gait practice; Gait pattern re-education; Functional stepping	Exercises to improve gait symmetry, cadence, step length, and lower extremity coordination	2–3 sets × 10–12 repetitions or 2–3 gait passes	Exhibits improved gait symmetry and stable foot clearance	Increase walking distance; increase speed; add dual-task challenges
F. Stretching	Hamstring stretch; Hip flexor stretch; Pectoral stretch; Wrist flexor stretch	Flexibility of major muscle groups prone to shortening in post-stroke populations	2–3 × 20–30 sec per muscle	Full passive stretch achievable without discomfort	Increase holding time; progress to active stretching; include proprioceptive neuromuscular facilitation (PNF)
